# Case Report: Facial fracture sequelae: the importance of using a specific customized implant (PSI) for orbital reconstruction

**DOI:** 10.3389/fsurg.2024.1425905

**Published:** 2024-10-25

**Authors:** Bianca Pulino, Leonardo Alckmin Hotz Fonseca, Eduardo Hochuli Vieira, José Roberto Piteri Filho, Thiago Felippe Oliveira de Mâcedo, Marcelo Pigatto D'Amado, Rodrigo Pereira, Igor Alexandre Damasceno Santos, Robert Ilesan, Henrique Cabrini Moreira, Drielli Viana, Raphael Capelli Guerra

**Affiliations:** ^1^ Department of Oral and Maxillofacial Surgery, Sírio Libanes Teaching and Research Institute, São Paulo, Brazil; ^2^ Department of Oral and Maxillofacial Surgery, Leforte/Dasa Hospital, São Paulo, Brazil; ^3^ Department of Oral and Maxillofacial Surgery, São Paulo State University, São Paulo, Brazil; ^4^ Sírio Libanes Teaching and Research Institute, São Paulo, Brazil; ^5^ Department of Oral and Maxillofacial Surgery, Grande Rio University - UNIGRANRIO, Rio de Janeiro, Brazil; ^6^ Department of Oral and Maxillofacial Surgery, Universidade Federal da Bahia, Bahia, Brazil; ^7^ Department of Craniomaxillofacial surgery, University Hospital Basel, Basel, Switzerland; ^8^ Municipal Hospital Doutor Arthur Ribeiro de Saboya, São Paulo, Brazil; ^9^ Department of Oral and Maxillofacial Surgery, Universidade Nove de Julho, São Paulo, Brazil

**Keywords:** orbital fracture, reconstruction, 3D printed, titanium implants, custom-made implants

## Abstract

The reconstruction of orbital fracture sequelae is a major challenge due to concerns regarding surgical approach and implant stability. Few anatomical sites of such minute size have presented with as much variation in treatment as the orbital floor fractures and related sequelae. Our patient developed sequelae of an orbital fracture over the last 3 years, presenting with dystopia, ophthalmoplegia, and diplopia in the supra- and lateroversion and aesthetic impairment. The variety of implant materials for reconstruction after orbital fractures is extensive, and the decision as to which material to use continues to be debated. The continuing development of computer-aided diagnosis and management and the construction of stereolithographic models offer comparable reproduction of anatomical detail. This technology is described in relation to the planning of trauma surgery and sequelae and the planning of ablative surgery for malignant neoplasms of the head and neck. The use of specific 3D printed titanium implants for bone defects was first reported in cranial reconstruction in 2012, and several studies have reported their use in orbital fractures. The advantages of this implant were increased stiffness, preventing shape loss during placement, a precise fit, and decreased surgical time. However, in the existing literature, the one-piece implant done in this way was a precise fit; therefore, it is possible that navigation between intraoperative anatomical landmarks is lost. However, in cases where reconstruction is difficult, such as extensive orbital wall fractures and large orbital sequelae, the 3D printed implant has been helpful in decreasing surgical time and can be accessed by a limited surgical approach with a precise fit. Our clinical case involved a 37-year-old male patient who experienced severe physical aggression in 2020, amid the COVID-19 pandemic. At the time, due to the overwhelming healthcare demands and resource constraints imposed by the pandemic, immediate surgical intervention for the correction of the fracture was not feasible. As a result of this delay, the patient developed sequelae of the orbital fracture over the last 3 years.

## Introduction

Orbital fracture sequelae pose a significant challenge for even seasoned surgeons due to the intricate anatomy and the critical need to harmonise the appropriate surgical approach with the optimal implant selection. Inadequate fitting of implants and suboptimal surgical techniques can result in visual impairments and aesthetically displeasing results. Orbital floor and wall fractures present considerable challenges due to the tricky three-dimensional (3D) anatomy and restricted operative visibility.

This often results in an increase or decrease of orbital volume that can cause asymmetrical positioning of the eyeball (dystopia), further complicating the clinical outcome, as presented in the following case. The management of these fractures necessitates meticulous dissection to the posterior margin and accurate reconstruction of the slope of the orbital floor. These steps are seen as essential for preventing residual enophthalmos and the recurrence of diplopia. The extent of the fracture and the volume of herniated soft tissue have been correlated with the delayed onset of enophthalmos.

Today, we have implantable materials such as titanium meshes, pre-moulded titanium meshes and customizations through implant prostheses specific to each patient. All of them have their benefits and few contraindications, most of which are due to the bone defect and the cost of the specific implant.

Custom-made implants, also known as patient-specific implants (PSIs), have contributed immensely to addressing many of these challenges, enhancing the precision and effectiveness of surgery. Almost 4 decades ago, in 1984, Charles W. “Chuck” Hull filed US patent number US4575330 A for “Apparatus for production of three-dimensional objects by stereolithography” (1). In his patent, he described the first modern 3D printer. Additive manufacturing or 3D printing has evolved in tandem with computing power, adhering quite closely to Moore's Law to this day, with consistently declining costs of computer-assisted design and manufacturing (CAD/CAM) technologies.

By the late 1990s, the first follow-up studies on the application of CAD/CAM in cranio-maxillofacial and neurosurgery were already being published, captivating the readers of renowned specialty journals (2, 3). The application of 3D technologies in medicine had evolved from simple bending (4, 5) to drilling procedures (6) and comprehensive 3D printing, encompassing various aspects of the medical field.

## Case description

Our clinical case involved a 37-year-old male patient who experienced severe physical aggression in 2020, amid the coronavirus disease 2019 pandemic. This altercation resulted in a complex right orbital fracture. At the time, due to the overwhelming healthcare demands and resource constraints imposed by the pandemic, immediate surgical intervention for the correction of the fracture was not feasible.

**Figure 1:**
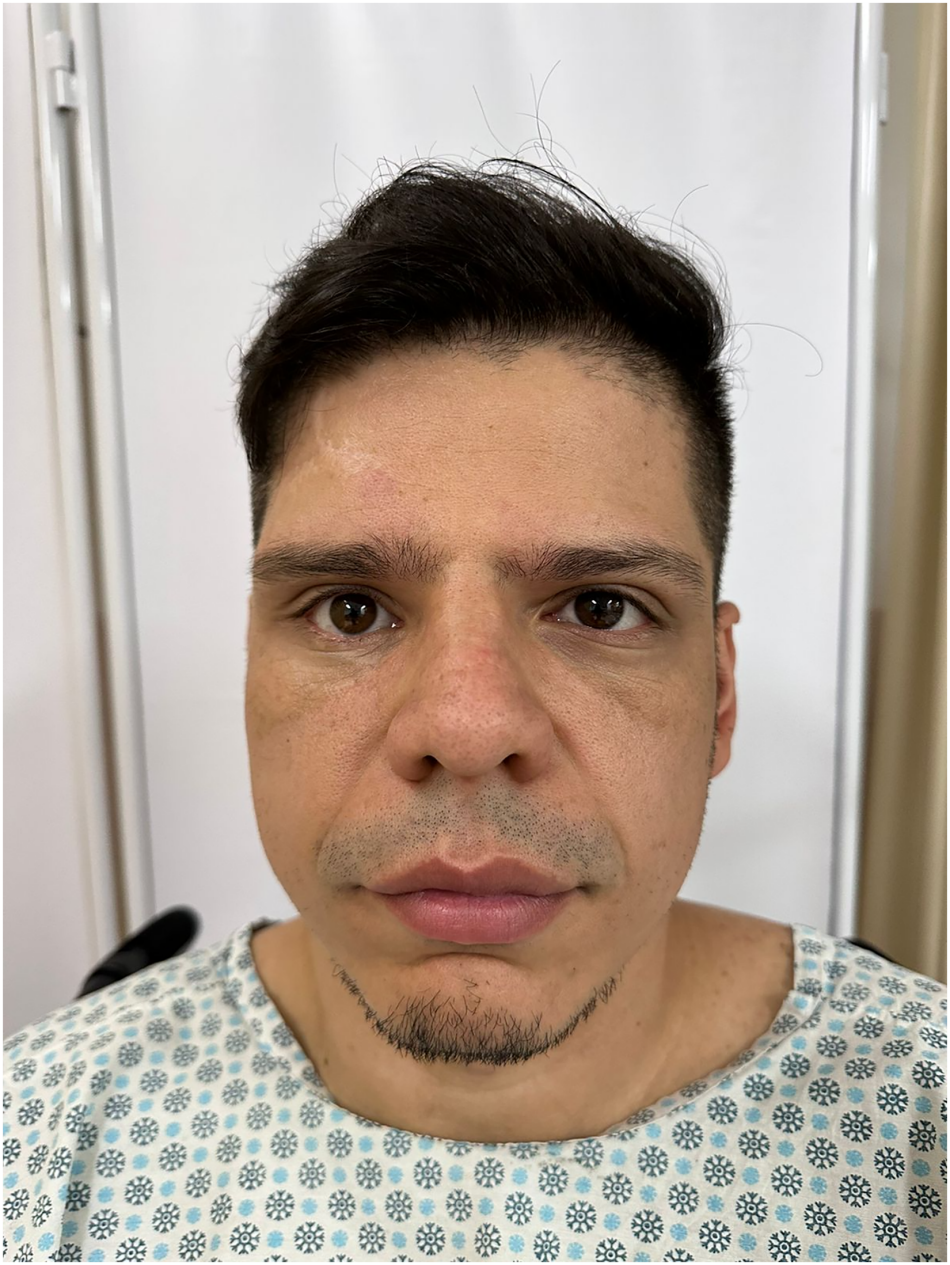
Preoperative view presenting with dystopia, ophthalmoplegia as well as aesthetic impairment.

As a result of this delay, the patient developed sequelae of the orbital fracture over the last 3 years, presenting with dystopia, ophthalmoplegia, and diplopia in the supra- and lateroversion and aesthetic impairment (Figure 1). According to the patient's report, the ophthalmologic symptoms presented in this trauma were difficulty moving the eyes and mild diplopia after the trauma, as well as signs of enophthalmos. The patient was in other trauma unit.

The surgical procedure at the time of the trauma was suspended due to the covid pandemic, and after 3 years the patient returned to our hospital to treat his complaints, which were restricted eye movement, facial aesthetics and altered positioning of the height of the eyeball (enophthalmos).

Due to the large sequelae defect, the only option to restore the contour and volume of the orbit was a customized titanium implant, knowing that conventional titanium mesh would not support or adapt to correct the defect.

**Figure 2:**
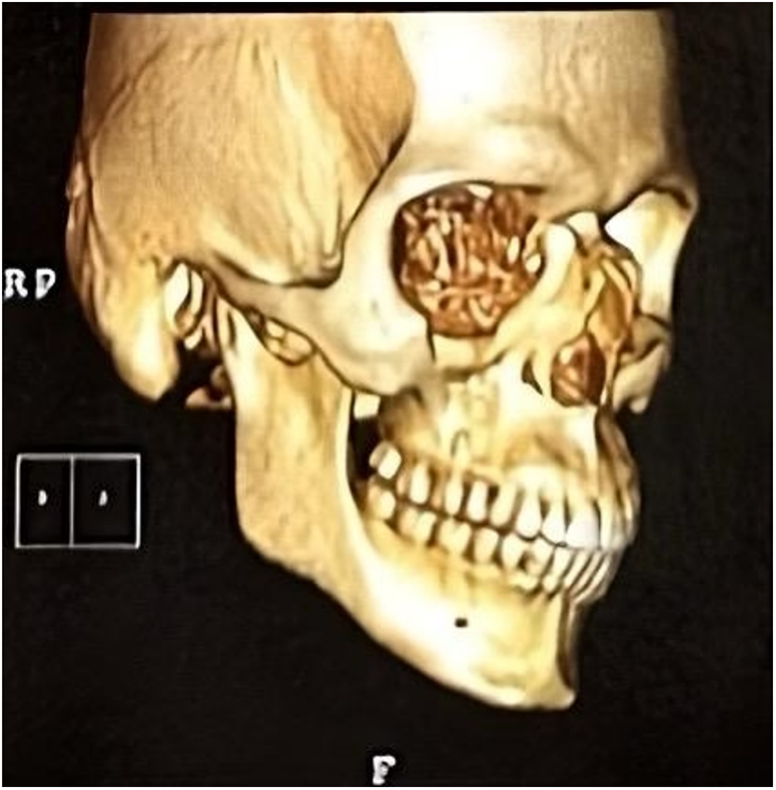
Computed tomography (CT) scan showing isolated medial and inferior orbital wall fractures.

Computed tomography of the face showed fracture traces compromising the anterior wall of the right maxillary sinus, associated with unevenness of the orbital floor, and fracture of the lamina papyracea and nasal bone (Figure 2). Initial management focused on stabilising the patient condition, paving the way for the implementation of a more innovative and tailored treatment strategy.

**Figure 3:**
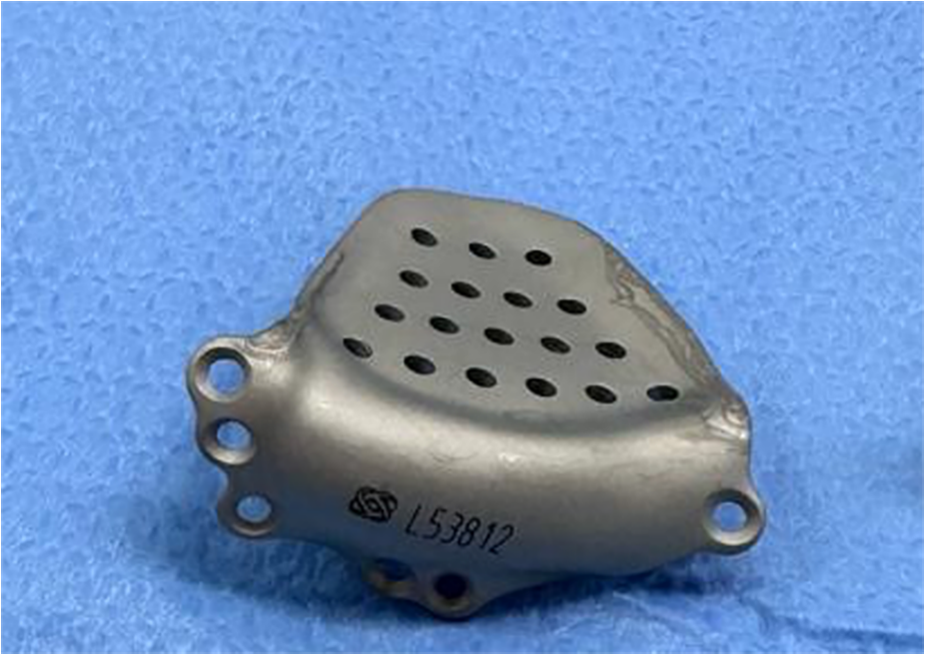
Custom-made titanium implant to orbital floor.

Faced with the severity of the orbital fracture and the limitations of standard reconstruction materials, we chose a PSI locally produced by Traumec®, (Rio Claro, Sao Paulo-based company/Brazil). This PSI, a tailor-made titanium prosthesis, was intricately designed to enable precise anatomical correction of the orbital fracture (Figure 3).

The measurements were collected using virtual planning software, CT scans to calculate the orbital volume and a mirror image of the contralateral orbit.

**Figure 4:**
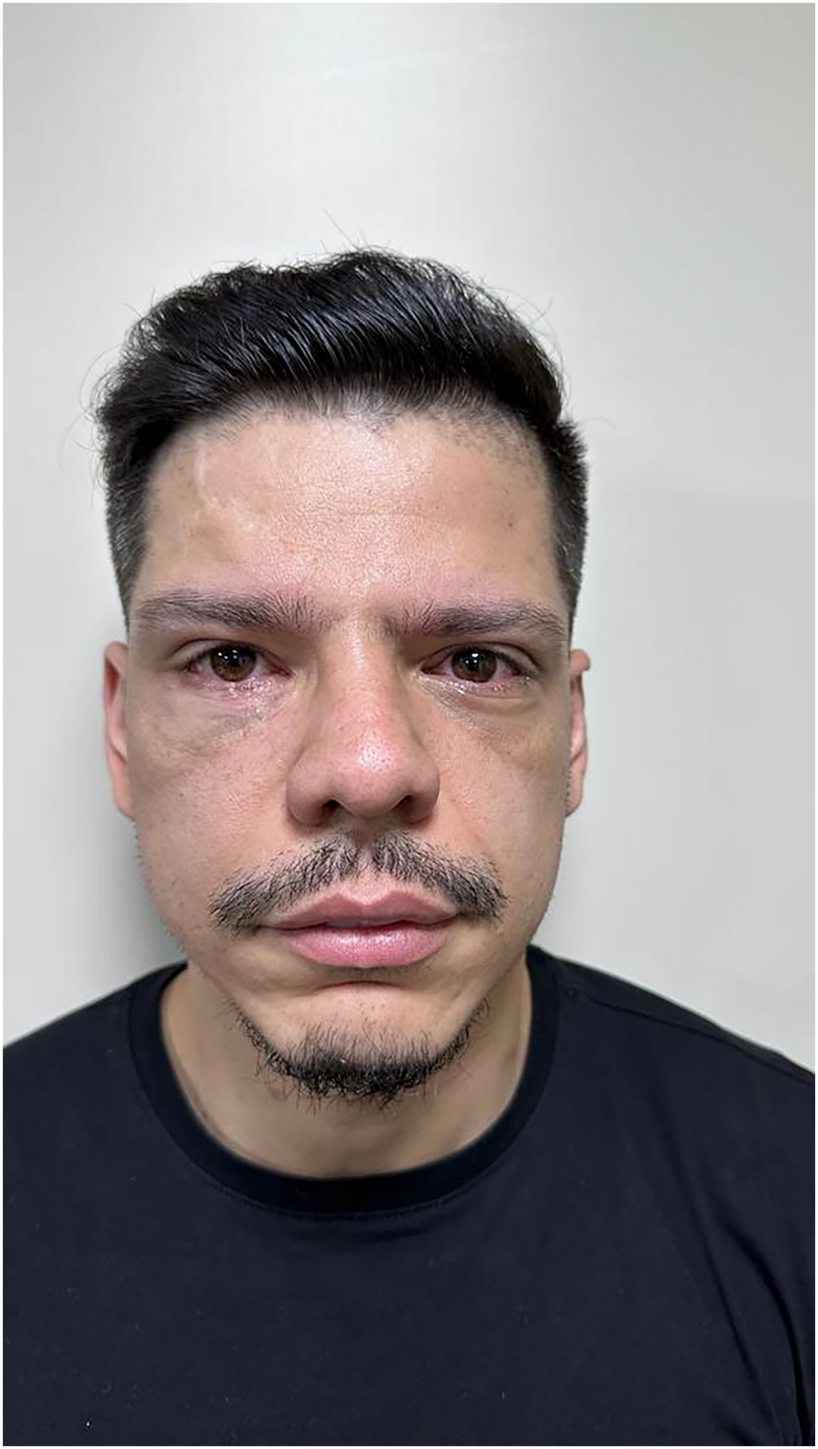
Positioning the customized prosthesis on the orbital floor.

The orbital reconstruction for the installation of the customised titanium prosthesis was carried out through the infraciliary surgical approach. It was possible to correct the ocular positioning and orbital perimeter without any impairment of ocular mobility and with adequate adaptation of the prosthesis (Figure 4).

The transconjunctival approach was not the first choice due to the limited surgical field for correct installation and adaptation of the customised prosthesis, which was why the intraciliary approach was chosen.

**Figure 5:**
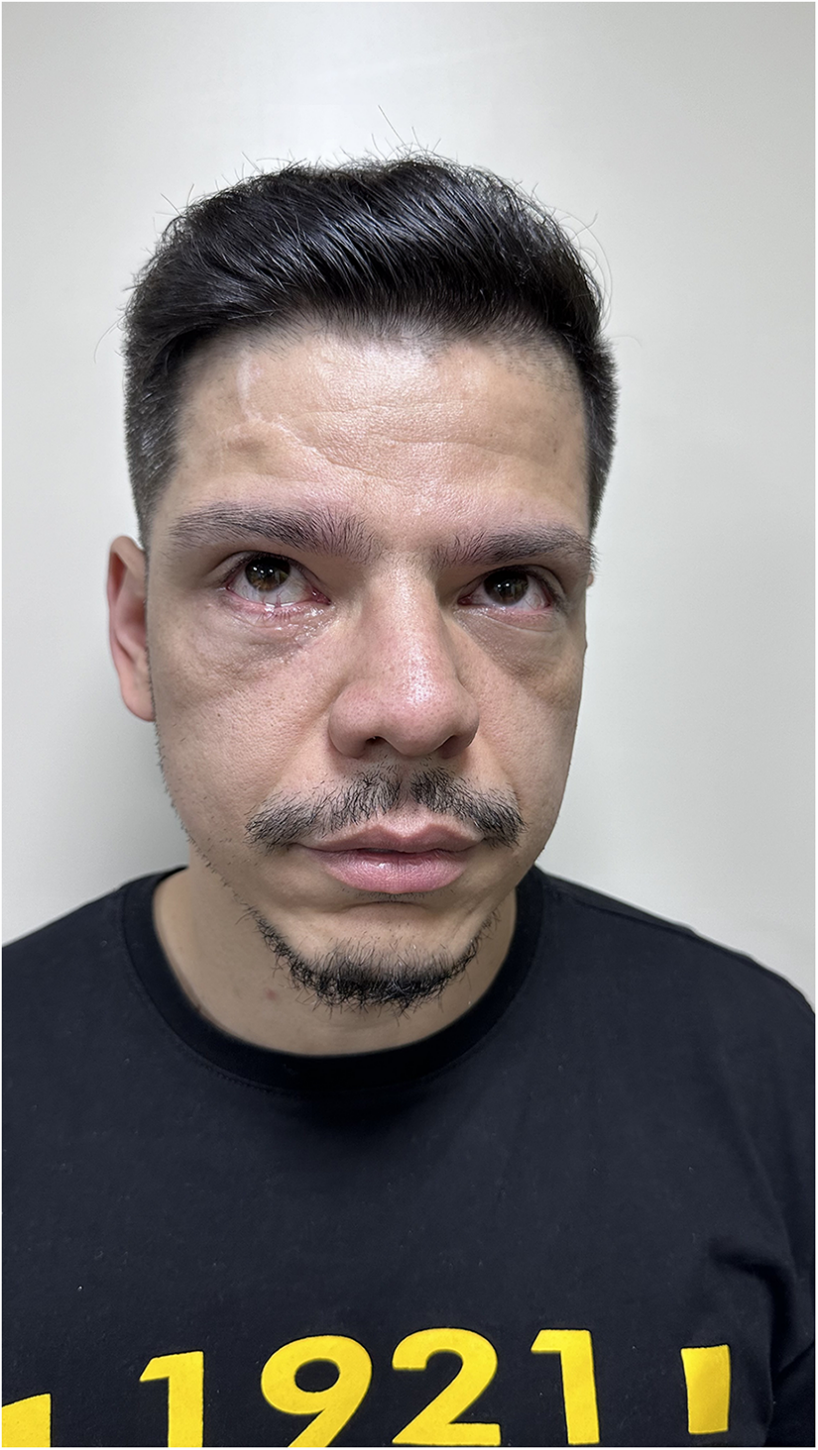
Pos operative to 6 months with no visual alterations, adequate ocular mobility, no diplopia, no ophthalmoplegia, adequate correction of the orbital perimeter and aesthetically satisfied.

**Figure 6:**
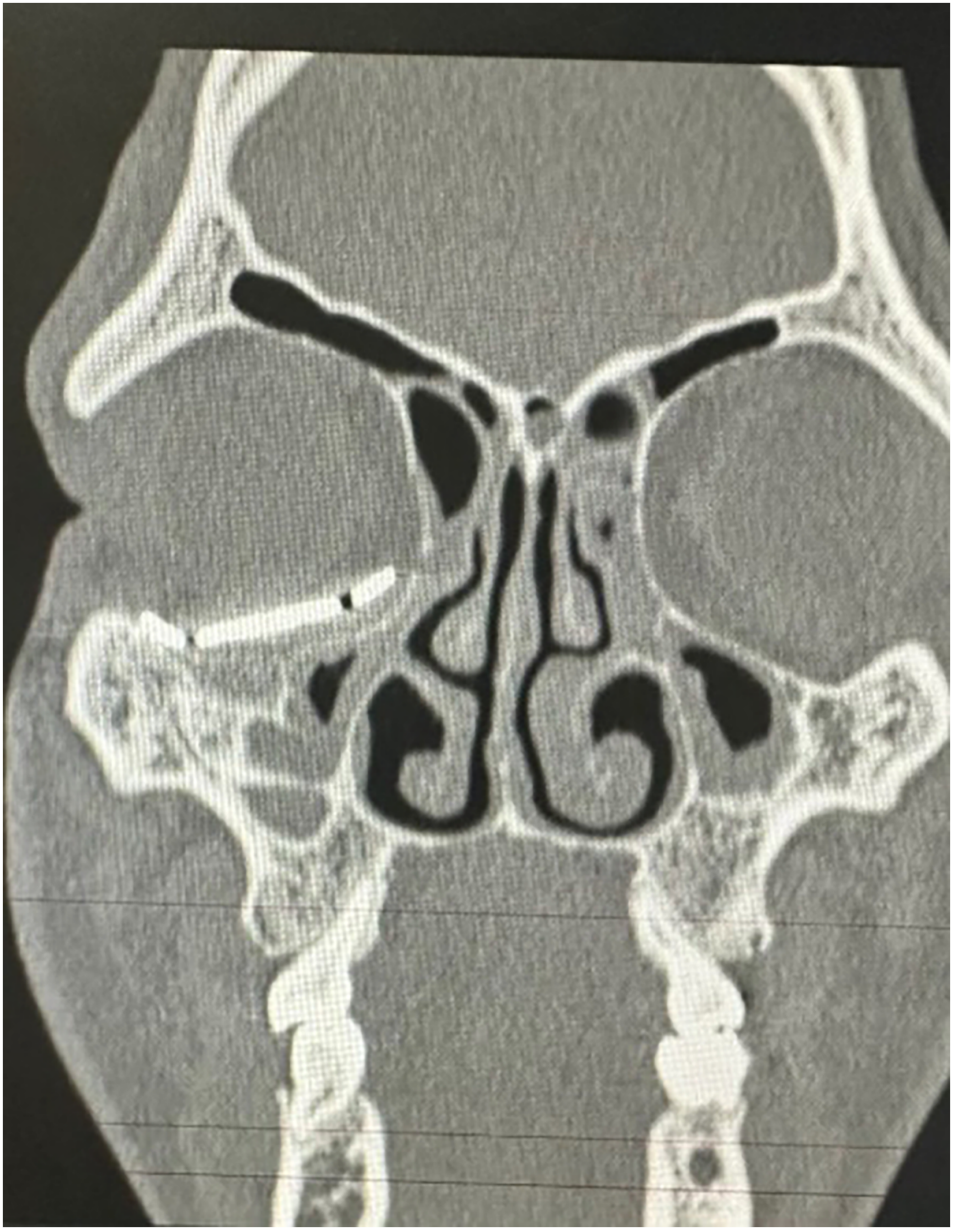
Coronal CT scan of the face showing the adaptation of the customized prosthesis.

**Figure 7:**
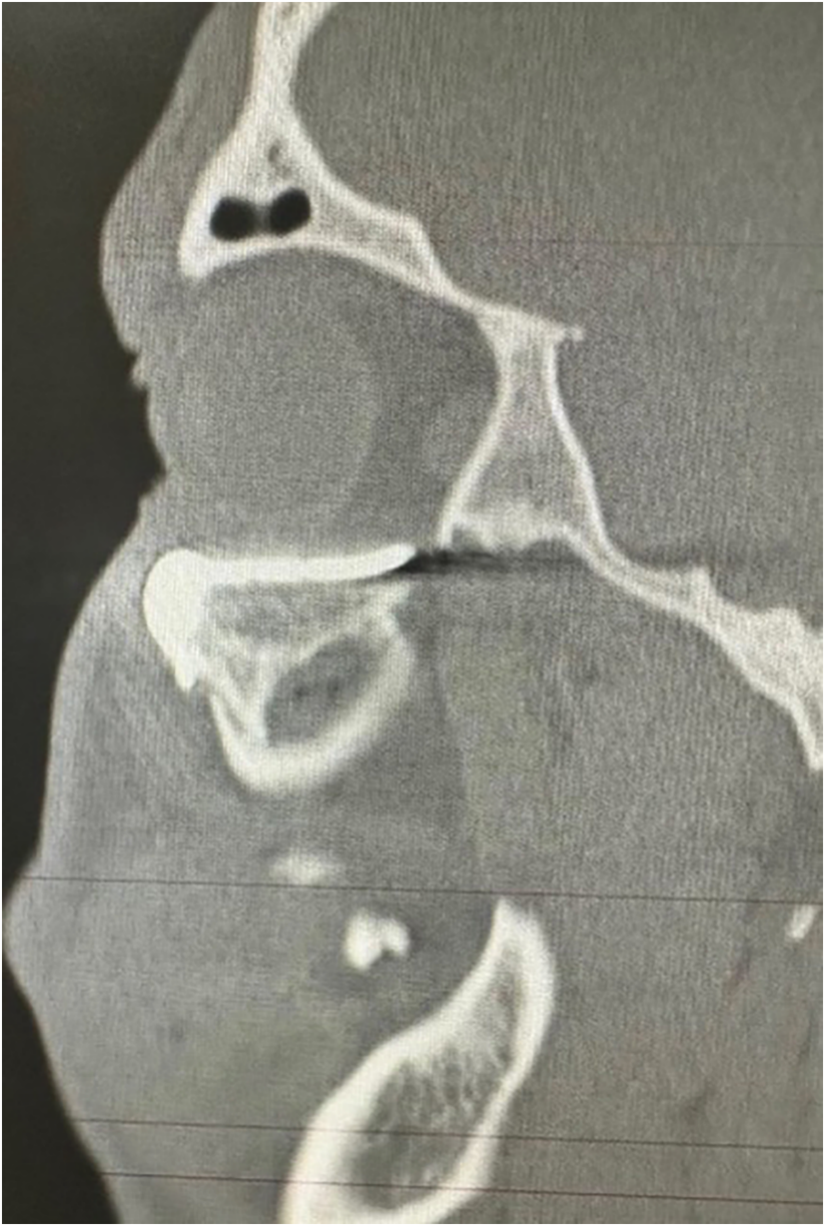
Sagital CT scan of the face showing the adaptation of the customized prosthesis.

The patient was followed up for 6 months, with no concerns of visual alterations, diplopia, and ophthalmoplegia and with adequate ocular mobility and correction of the orbital perimeter. He was also satisfied with the aesthetic result of the orbital reconstruction (Figures 5–7).

Its customisation played a crucial role in the accurate repositioning of the eyeball, effectively correcting the dystopia. This innovative approach not only demonstrated the efficacy of using advanced bespoke materials in craniofacial surgery but also highlighted the potential improvements in patient outcomes, combining functional restoration and aesthetic considerations.

## Discussion

Early reconstruction of the fracture (within 2 months) offers better results than those of delayed correction of diplopia and enophthalmos when already established, with scarred peribulbar tissue complicating the situation. Depending on the size of the defect, different materials can be used. Some suggest the use of absorbable synthetic implants, such as polyglactin and polytrioxane sheets, to cover small defects (7). The most commonly used materials for extensive defects are titanium, autogenous or allogenous bone grafts, and hydroxyapatite blocks (8). In recent years, with the development of machine processing, the use of new materials, such as bioceramic implants, has become an interesting alternative (9).

Titanium is the standard material used in craniofacial plate systems due to its high mechanical strength and resistance to corrosion. Titanium is highly biocompatible and non-ferromagnetic and has excellent durability and low weight, which makes it suitable for magnetic resonance imaging and yields low artifacts in computed tomography (10).

It has excellent stability and rigidity (with a modulus of elasticity higher than that of native bone), which makes it ideal for fixing craniofacial fractures and plating for augmentation purposes, especially in the aesthetic context. It can also be used for customisations (11). The greater mechanical strength and resistance to deforming forces can lead to a “stress shielding effect” in the adjacent bone, leading to a loss of structure and strength in the surrounding native bone. This effect can lead to unwanted loosening of the implant (12). The use of metal fixation in craniofacial surgery has been widely studied. Complication rates vary according to location, with plates used in mandibular reconstruction having the highest incidence of overall complications (14% infection rate, 20% need for plate removal (13). Despite its excellent biocompatibility, corrosion resistance, and strength, titanium can be visible through thin areas of the skin, particularly in the periorbital region. This can lead to undesirable aesthetic results (10, 14).

Other well-known issues of titanium implants and plates are thermal sensitivity/intolerance (due to higher thermal conductivity than that of surrounding tissues, which can lead to an unpleasant cold feeling) and discomfort, often resulting in implant removal (15).

Autologous materials reportedly offer clear advantages with cartilage, calvaria bone, and iliac bone. These grafts offer uncertain longevity and cause morbidity at the donor site. Alloplastic materials have the longest history of use, albeit with a well-documented complication rate related to graft extrusion. Other alloplastic materials, such as polyethylene sheet (Med pore), demonstrate satisfactory results. Newer absorbable materials, such as polydioxanone, are another option. The role of bioactive glass has been reported more recently, but its use is limited by the size of the defect. Titanium, on the other hand, is an inert and widely used material, but in its preformed form, it can be cumbersome to use on the orbital floor. It also poses a surgical challenge in cases of removal (16).

We currently have different shapes and sizes of implantable titanium materials available, such as ordinary titanium meshes, pre-moulded meshes and customized meshes, which are prostheses developed specifically for each patient. All of them have benefits in common, such as the material's compatibility in facial reconstructions, which has already been strongly established in the literature. Common meshes and pre-moulded meshes have the function of reconstructing the bone defect, but the surgeon's ability to adapt and visualize the correct stability and adaptation of the mesh is necessary to avoid post-operative complications. Customized prostheses have the advantages of prior planning, the security of perfect adaptation and the possibility of correcting the orbital volume, as in the clinical case described in this article, which other materials are unable to do, but their cost is higher due to their complexity.

The continuing development of computer-aided diagnosis and management and the construction of stereolithographic models offer comparable reproduction of anatomical detail (17). This technology is described in relation to the planning of trauma surgery and sequelae and of ablative surgery for malignant neoplasms of the head and neck. Custom imaging of the orbital floor is possible, although the choice of material is currently being debated (18).

The use of specific 3D printed titanium implants for bone defects was first reported in cranial reconstruction in 2012, and several studies reported their use in orbital fractures. The advantages of this implant were precise fitting, decreased surgical time, and increased stiffness, preventing shape loss during placement (19).

Recent literature has delved into the utilisation of two-piece 3D printed implants for treating combined orbital floor and medial wall fractures.

In 2016, Bio Architects®, a company based in Sao Paulo, in collaboration with the Swedish firm Arcam, received the first FDA certification to produce 3D printed titanium implants.

However, these variants may not fully exploit the capabilities of personalised implants, particularly in terms of structural cohesion and customisation, as effectively as one-piece implants do. The design of the latter ensures a precise fit. However, it introduces potential challenges in navigating intraoperative anatomical landmarks (20). A primary limitation of this approach is the associated time and cost: digital planning required 55 min, the manufacturing process spanned 10 h, and the delivery period extended over 3 working days. Despite these implants being more expensive compared to conventional flexible titanium plates and mesh, their utility becomes particularly pronounced in complex cases like extensive orbital wall fractures or significant orbital sequelae. Customised implants substantially reduced surgical time and permitted a limited surgical approach, while guaranteeing a precise fit. Of note, in cases involving orbital volume loss, these implants enable thorough planning, thereby restoring the position of the eyeball with improved functional and aesthetic results.

In our country, the high cost of making customised prostheses means that the use of this technology is exclusive to patients with health insurance. However, with the dissemination of satisfactory long-term results based on cohort studies and comparative analyses with traditional orbital reconstruction methods, this prosthesis customisation technology may become more accessible to surgeons.

## Conclusion

In conclusion, a point-of-care cost-benefit analysis revealed that personalised titanium implants, despite their initial cost, offer a predictable and superior correction of fracture sequelae. This approach significantly reduced surgical time, directly translating into decreased patient morbidity. Furthermore, the long-term durability and effectiveness of these implants underscored their value, promising not only immediate surgical improvements but also enhanced patient outcomes over time. This innovation in craniofacial surgery represents a pivotal step towards more efficient patient-centred care.

## Data Availability

The original contributions presented in the study are included in the article/Supplementary Material, further inquiries can be directed to the corresponding author.
